# The antifibrotic drug pirfenidone inhibits spondyloarthritis fibroblast-like synoviocytes and osteoblasts in vitro

**DOI:** 10.1186/s41927-018-0040-9

**Published:** 2018-11-19

**Authors:** Julie Stougaard, Søren Lomholt, Pernille Ommen, Jens Kelsen, Tue W. Kragstrup

**Affiliations:** 1 Department of Biomedicine, Aarhus University, Aarhus, Denmark; 2 Department of Dermatology, Aarhus University Hospital, Aarhus, Denmark; 3 Department of Hepatology and Gastroenterology, Aarhus University Hospital, Aarhus, Denmark; 4 Department of Rheumatology, Aarhus University Hospital, Aarhus, Denmark; 5 Department of Internal Medicine, Randers Regional Hospital, Randers, Denmark

**Keywords:** Spondyloarthritis, Inflammation, Fibroblast, Myofibroblast, Osteoblast, Pirfenidone, Fibrosis

## Abstract

**Background:**

The pathogenesis of spondyloarthritis (SpA) involves both inflammation and new bone formation in the spine. In line with this, the disease has been characterized as both inflammatory and fibrotic. The current treatment dampens inflammation while new bone formation can progress. Therefore, there is an unmet therapeutic need for the treatment of new bone formation in SpA. Fibrosis is mediated by myofibroblasts and new bone formation is the result of increased osteoblast mineralization and decreased osteoclast resorption. Here, we evaluate the potential effect of the newly approved anti-fibrotic agent pirfenidone (PFD) on fibrosis and new bone formation in cell culture models of SpA.

**Methods:**

Fibroblast-like synoviocytes (FLSs) were isolated from SpA patients (*n* = 6) while the osteoblast cell line Saos-2 was purchased. The cells were cultured with PFD at 0.25 0.5, or 1.0 mg/ml. The proliferation of FLSs was analyzed with light microscopy and flow cytometry. The differentiation and activation of FLSs was assessed with flow cytometry, a membrane-based antibody array and enzyme-linked immunosorbant assays. The mineralization capacity of osteoblasts was studied with an assay measuring deposition of hydroxyapatite.

**Results:**

PFD reduced the Ki67 expression 7.1-fold in untreated FLSs (*p* = 0.001) and 11.0-fold in FLSs stimulated with transforming growth factor beta (TGFβ), tumor necrosis factor alpha (TNFα), and interferon gamma (IFNγ) (*p* = 0.022). There were no statistically significant changes in membrane expression of alpha smooth muscle actin (αSMA), intercellular adhesion molecule 1 (ICAM-1), or human leukocyte antigen DR (HLA-DR). In supernatants from FLSs stimulated with TGFβ, TNFα, and IFNγ, PFD decreased the secretion of 3 of 12 proteins more than 2-fold in the membrane-based antibody array. The changes in secretion of monocyte chemoattractant protein 1 (MCP-1) and chitinase-3-like protein 1 (CHI3L1, YKL-40) were validated with ELISA. PFD decreased the secretion of both Dickkopf-related protein 1 (DKK1) (*p* = 0.006) and osteoprotegerin (OPG) (*p* = 0.02) by SpA FLSs stimulated with TGFβ, TNFα, and IFNγ. Finally, PFD inhibited the deposition of hydroxyapatite by osteoblasts in a dose-dependent manner (*p* = 0.0001).

**Conclusions:**

PFD inhibited SpA FLS proliferation and function and osteoblast mineralization in vitro. This encourages studies of the in vivo effect of PFD in SpA.

## Background

Spondyloarthritis (SpA) is characterized by inflammation of the axial skeleton and includes ankylosing spondylitis, reactive arthritis, psoriatic arthritis and arthritis associated with inflammatory bowel disease [1, 2]. The pathogenesis of SpA involves both inflammation and new bone formation in the spine and the disease has been characterized as both inflammatory and fibrotic [3, 4]. The inflammatory component causes pain and morning stiffness and can be managed with non-steroidal anti-inflammatory drugs and inhibitors of tumor necrosis factor alpha (TNFα), interleukin 17 (IL-17) and IL-23. The calcification of the tendons and ligaments and new bone formation leading to ankylosis of the spine causes irreversible compromised range of motion. The retardation of ankylosis has still not been successful [5, 6]. The role of non-steroidal anti-inflammatory drugs still has to be defined, early and long term treatment seems to be necessary for the TNFα inhibitors and the role of blocking IL-17 and IL-23 is still not clear [7]. Therefore, there is an unmet therapeutic need for the treatment of new bone formation in SpA.

The inflammation in SpA is not fully understood but involves bacterial and mechanical stress [1, 8]. The most prominent immune abnormality in SpA is the genetic association with human leukocyte antigen B27 (HLA-B27). Further, several proinflammatory membrane molecules with importance for cell adhesion and immune activation are upregulated including intercellular adhesion molecule 1 (ICAM-1). Finally, several cytokines and chemokines are important in the pathogenesis of SpA including macrophage derived TNFα, and lymphocyte derived interferon gamma (IFNγ) and IL-17 [9], while other molecules have been suggested as biomarkers of diagnosis or treatment response such as chemoattractant protein 1 (MCP-1, CCL2) and chitinase-3-like protein 1 (CHI3L1, YKL-40) [10, 11].

Fibrosis is mediated by alpha smooth muscle actin (αSMA) expressing myofibroblasts as seen in wound healing and fibrotic diseases such as systemic scleroderma and lung fibrosis [12, 13]. One of the distinct features discriminating spondyloarthritis from rheumatoid arthritis is transforming growth factor beta (TGFβ) induced upregulation of myofibroblasts involved in new bone formation at entheseal sites [4, 14]. Bone metabolism is otherwise the result of increased osteoblast mineralization and decreased osteoclast resorption. A balance between activating bone morphogenetic proteins and inhibiting Dickkopf-1 (DKK1) regulates osteoblast activity. Osteoclasts are activated by the interaction between receptor activator of nuclear factor-κB (RANK) on osteoclasts and RANK ligand (RANKL), which is blocked by osteoprotegrin (OPG).

Because new bone formation in SpA resemble fibrosis in fibrotic diseases these processes could have similar therapeutic targets and share treatment approaches [3]. Pirfenidone (PFD, brand names Esbriet and Pirespa) is a new drug used to treat idiopathic lung fibrosis. It is an orally active small molecule (MW 185) that is able to move through cell membranes without requiring a receptor. The drug is relatively well tolerated [15, 16]. PFD has been approved for the treatment of idiopathic lung fibrosis [17]. Further, PFD has shown promising effects in several animal models and in clinical trials of other fibrotic diseases and in a small cohort of patients with rheumatoid arthritis [15, 16, 18, 19].

Here, we evaluate the potential effect of PFD in cell culture models of SpA. We hypothesize that PFD inhibits the formation and activity of spondyloarthritis myofibroblasts and osteoblasts and thereby potentially reduces new bone formation in spondyloarthritis.

## Methods

### Study subjects

A study population consisting of SpA patients (*n* = 6) with peripheral involvement was included for obtaining synovial fluid for growing fibroblast-like synoviocytes (FLSs). Patients with peripheral arthritis contacted the clinic because of a knee joint effusion. No disease activity or prognosis scores or test results were obtained for this study population.

### Sample handling

Synovial fluid mononuclear cells (SFMCs) were isolated by conventional Ficoll-Paque (GE Healthcare) density-gradient centrifugation and cryopreserved at **−** 135 °C until time of use.

### Fibroblast-like synoviocyte cultures

FLSs were grown from SFMCs as described previously [20, 21]. Briefly, SFMCs were thawed and cultured in Dulbecco’s modified Eagle medium (Lonza) supplemented with 10% fetal bovine serum (FBS), penicillin, streptomycin, and glutamine at 37 °C and 5% CO_2_ at a density of 2 × 10^6^ cells/ml in a humidified incubator, replacing the medium every 3 to 4 days. When the cell layer was 70% confluent, the FLSs were passaged by trypsin treatment and used for analyses at passage 4–5. FLSs were stimulated with either TGFβ at 5 ng/ml, TNFα at 10 ng/ml, and IFNγ at 10 ng/ml alone or in combination and cultured with or without PFD at 1.0 mg/ml for 48 h. This concentration has previously been shown not to induce apoptosis or cell death in fibroblasts from several anatomical sights including FLSs [22–25]. Supernatants were harvested and kept at − 80 °C until time of use.

### Flow cytometry

SpA FLSs were cultured at a density of 5.0 × 10^4^ cells/ml in RPMI-1640 (Lonza) supplemented with 10% FBS, penicillin, streptomycin, and glutamine at 37 °C and 5% CO_2_ in a humidified incubator for 24 h. Cells were stimulated with either TGFβ at 5 ng/ml, TNFα at 10 ng/ml, and IFNγ at 10 ng/ml alone or in combination and cultured with or without PFD at 1.0 mg/ml for 48 h. Cells were then harvested by trypsin/EDTA treatment, transferred to polypropylene tubes (Nunc) and fixed using 4% formaldehyde (Sigma Aldrich) diluted in PBS. Cells were then permeabilized using 0.3% saponin (Sigma Aldrich) in PBS with 0.5% bovine serum albumin (BSA) (Calbiochem) and 0.09% NaN_3_. Staining with antibodies was done in a buffer containing 10 μg/ml mouse gamma globulin (Jackson ImmunoResearch) to minimize non-specific binding [26]. Cell surface staining before permeabilization was done with anti-CD90 phycoerythrin cyanine 7 (PC7) (BioLegend), anti-ICAM-1 allophycocyanin (APC) (BD), and anti-HLA-DR phycoerythrin (PE) (BD) with incubation 30 min at 4 °C. Intracellular staining after permeabilization was done with anti-Ki67 alexa488 (BioLegend) and anti-αSMA APC (R&D Systems) with incubation 30 min at 4 °C. Dead cells were excluded based on staining with Live/Dead fixable viability marker (near-infra red, Life Technologies). The samples were analysed using an LSR Fortessa flow cytometer (BD Biosciences) and data analysed using FlowJo software version 10 (Tree Star Inc.).

### Membrane-based antibody array

Culture supernatants were analysed with a membrane-based antibody array for the parallel determination of the relative levels of cytokines and chemokines as done previously (Proteome Profiler™ Human XL Cytokine Array Kit, R&D Systems) [27].

### Enzyme linked immunosorbant assay

Culture supernatants were analysed with a commercially available MCP-1 (Biolegend), YKL-40 (R&D Systems), RANKL (R&D Systems), DKK-1 (R&D Systems), OPG (R&D Systems) enzyme-linked immunosorbant assays (ELISAs) following manufacturer’s instructions.

### Osteoblast cultures

The osteoblast cell line Saos-2 was used. The cells were cultured and expanded in supplemented osteoblast growth medium (C-27001, PromoCell). The cells were seeded in triplicates in 96-well plates at a concentration of 100,000 cells/ml (20,000 cells/well). Cells were then cultured in osteoblast mineralization medium (C-27020, PromoCell) with PFD either at 0.25 mg/ml, 0.5 mg/ml, or 1.0 mg/ml for 14 days. A negative control culture without PFD was used in each experiment for comparison. Medium with mediators was changed every 2–3 days. On day 14 the formed mineral was visualized using a commercial mineralization stain kit (OsteoImage, PA-1503, Lonza), which conjugates a fluorophore to hydroxyapatite in the mineral. Finally, the degree of mineralization was quantified using a plate reader (Thermo Scientific, Fluoroscan Ascent FL) [28, 29].

### Statistics

All flow cytometry, ELISA and mineralization measurements and clinical scores were expressed with the median and interquartile range (IQR). Flow cytometry, ELISA and mineralization measurements ratios were log-transformed and comparisons were made with the paired t-test or the repeated measures one-way ANOVA depending on the number of groups. A two-tailed *P*-value below 0.05 was considered statistically significant. Calculations and graphs were made with Stata (StataCorp LP) and GraphPad Prism (GraphPad Software).

## Results

### Proliferation of SpA FLSs

**Fig. 1:**
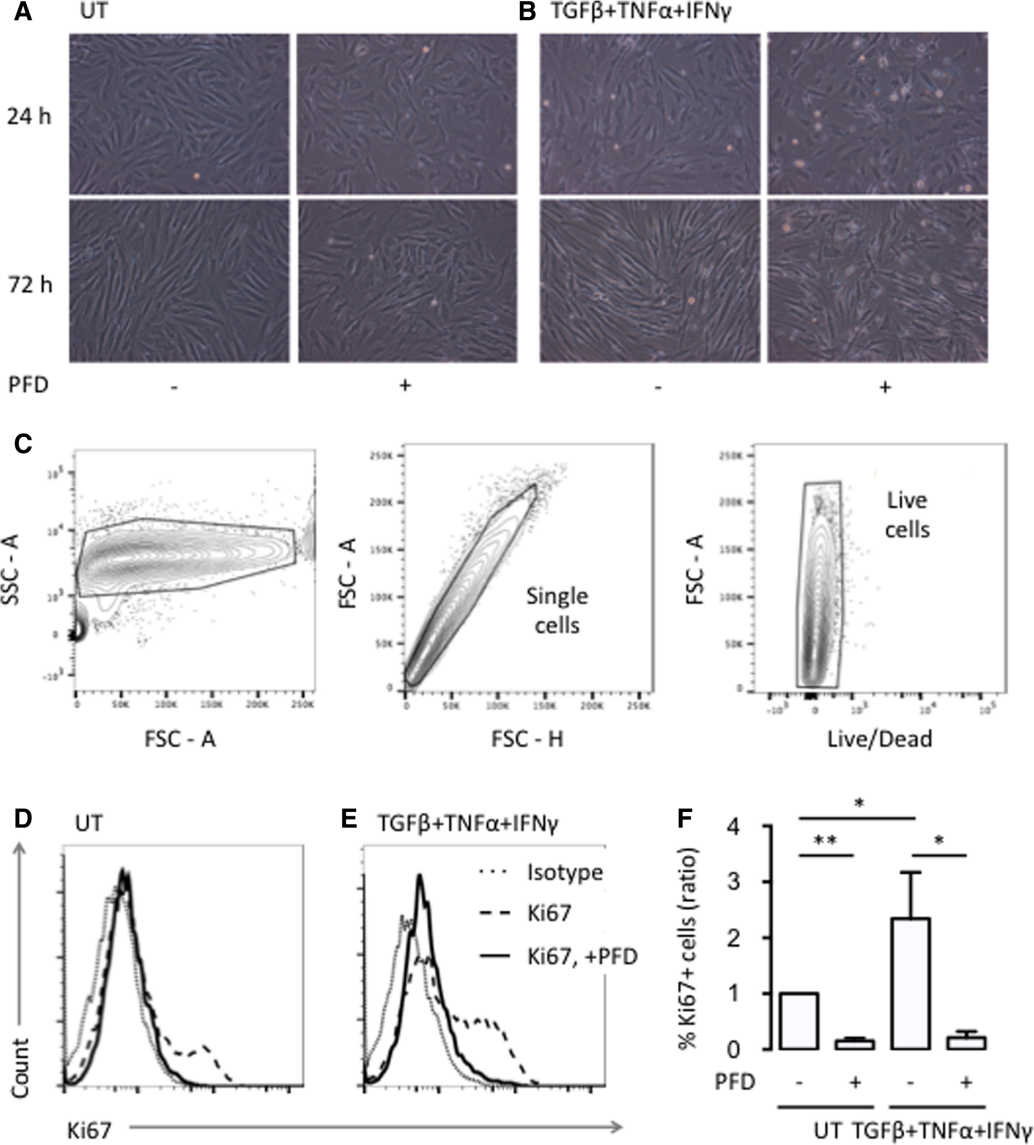
Effects of PFD on proliferation of SpA FLSs. **a**-**b** Representative light microscopy images of SpA FLSs untreated (UT) and stimulated (TGFβ+TNFα+IFNγ) with or without pirfenidone (PFD). **c** Representative flow cytometry plots of stimulated FLS treated with pirfenidone (PFD) showing the gating strategy. **d**-**e** Representative flow cytometry histograms of Ki67 expression in SpA FLSs untreated (UT) and stimulated (TGFβ+TNFα+IFNγ) with or without PFD. Isotype antibody was used as a negative control of the Ki67 staining. **f** Column bar graph of percentage of Ki67 positive FLSs among all SpA FLSs untreated (UT) and stimulated (TGFβ+TNFα+IFNγ) with or without PFD (*n* = 3). Data were normalized to untreated cultures (ratio), log-transformed and analyzed with the paired t-test. Boxes and bars indicate median and IQR. * *p* < 0.05. ** *p* < 0.01

We first evaluated the effect of PFD on fibroblast proliferation. PFD decreased the number of fibroblasts after 24 h and 72 h of culture without causing detachment of cells as evaluated by light microscopy (Fig. 1a and b). This was seen in both untreated cells and cells stimulated with TGFβ, TNFα, and IFNγ. The median percentage of dead cells measured with the Live/Dead fixable viability marker by flow cytometry was 0.31% (IQR 0.010% to 0.57%) for untreated cells and 0.20% (IQR 0.035% to 0.28%) for pirfenidone treated cells (Fig. 1c). The decreased proliferation was quantified by measuring Ki67 expression with flow cytometry (Fig. 1d and e). PFD reduced the Ki67 expression 7.1-fold in untreated cells (*p* = 0.001) and 11.0-fold in cells stimulated with TGFβ, TNFα, and IFNγ (*p* = 0.022) (Fig. 1f).

### Expression of intracellular αSMA and membrane HLA-DR and ICAM-1 in SpA FLSs

**Fig. 2:**
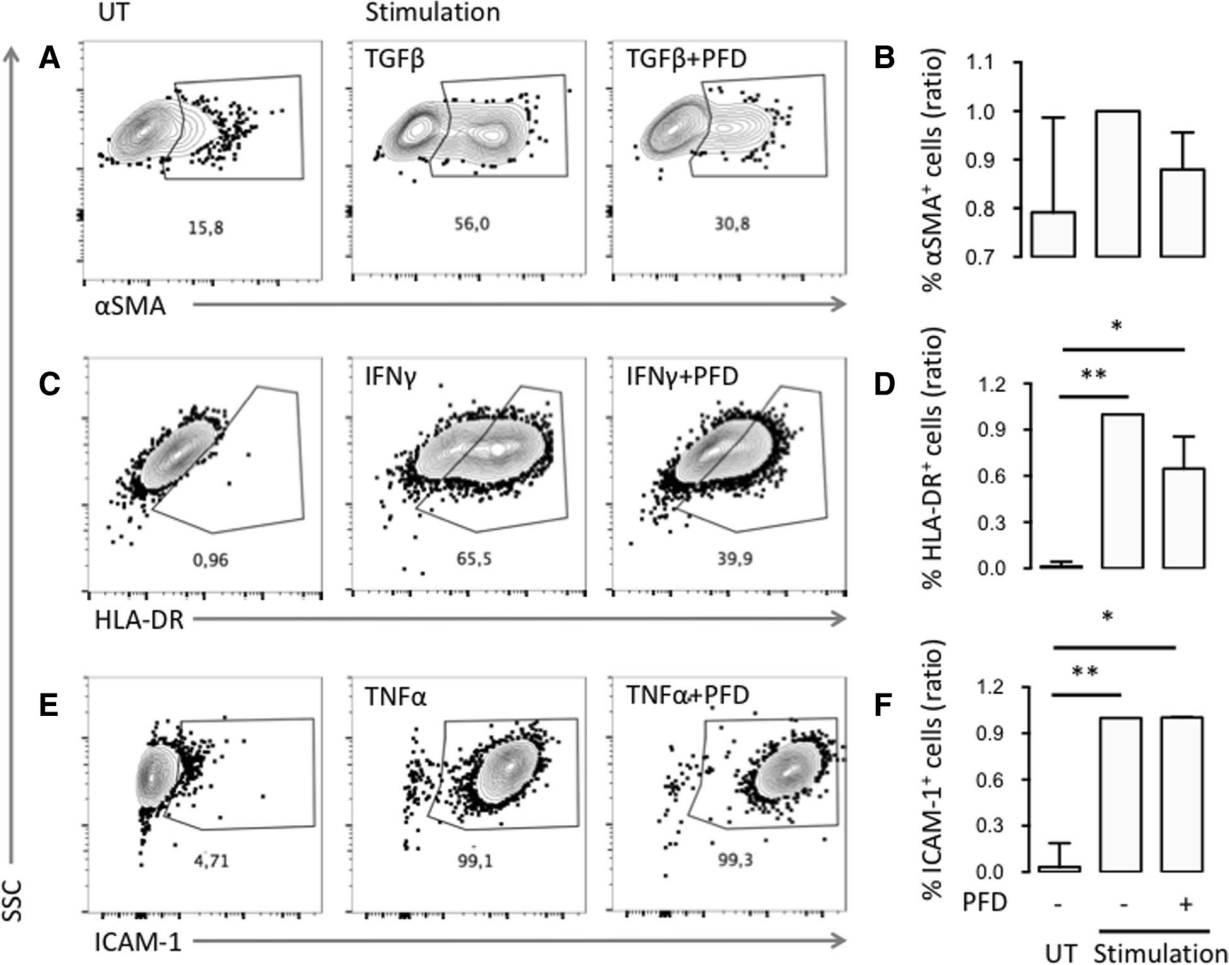
Effects of PFD on intracellular and membrane molecules characterizing differentiation and activity of SpA FLSs. **a**, **c**, and **e** Representative flow cytometry density plots of SpA FLSs untreated (UT) and stimulated (Stimulation) with or without pirfenidone (PFD). **b**, **d**, and **f** Column bar graph of percentage positive FLSs among all SpA FLSs untreated (UT) and stimulated (Stimulation) with or without PFD (*n* = 4). Data were normalized to stimulated cultures without PFD (ratio), log-transformed and analyzed with the paired t-test. Boxes and bars indicate median and IQR. **a** and **b** TGFβ stimulated FLSs were analyzed for intracellular αSMA expression. **c** and **d** IFNγ stimulated FLSs were analyzed for membrane HLA-DR expression. **e** and **f** TNFα stimulated FLSs were analyzed for membrane ICAM-1 expression. * *p* < 0.05. ** *p* < 0.01

We now studied whether PFD could alter expression of intracellular and membrane molecules characterizing FLS differentiation and activity. We studied the induction of three molecules known to be induced by IFNγ, TNFα, and TGFβ. These were the major histocompatibility complex HLA-DR, ICAM-1, and αSMA, respectively. The TGFβ induced upregulation of αSMA (Fig. 2a and b) and the IFNγ induced upregulation of HLA-DR (Fig. 2c and d) were not significantly decreased by PFD treatment. There was no difference between the percentage of ICAM-1 positive FLSs in TNFα stimulated cultures treated with or without PFD (Fig. 2e and f).

### Secretion of cytokines and chemokines from SpA FLSs

**Fig. 3:**
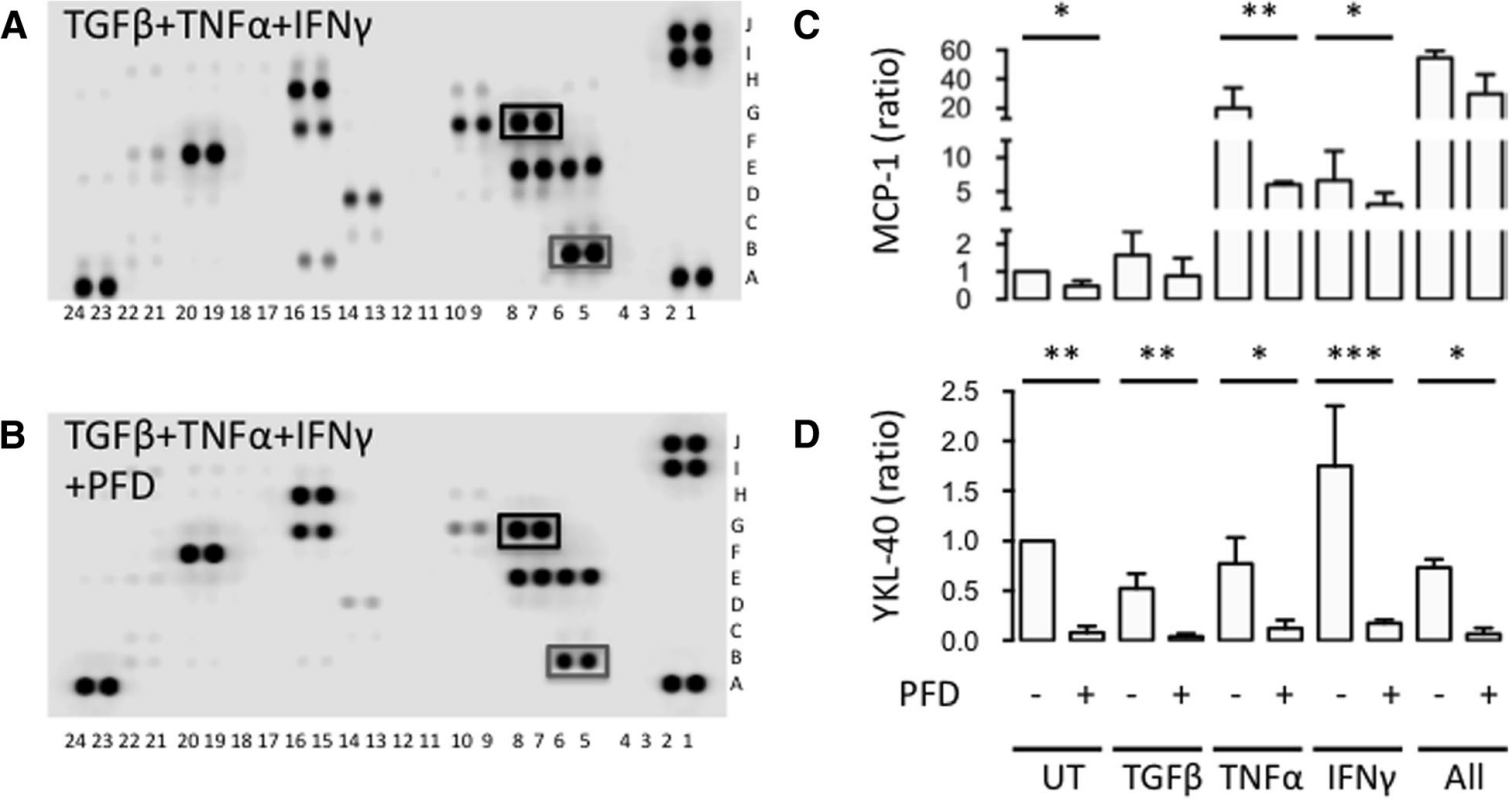
Effects of PFD on intracellular and membrane molecules characterizing differentiation and activity of SpA FLSs. **a**-**b** Images of membrane-based antibody array of SpA FLSs stimulated with TGFβ, TNFα, and IFNγ (TGFβ+TNFα+IFNγ) with or without pirfenidone (PFD). Black square marks MCP-1 and grey square marks YKL-40. **c**-**d** Column bar graph of MCP-1 and YKL-40 secretion by SpA FLSs untreated (UT) and stimulated with TGFβ, TNFα, IFNγ or all three cytokines (All) with or without PFD (*n* = 4). Data were normalized to untreated cultures without PFD (ratio), log-transformed and analyzed with the paired t-test. Boxes and bars indicate median and IQR. * *p* < 0.05. ** *p* < 0.01. *** *p* < 0.005

We then examined the effect of PFD on the secretion of cytokines and chemokines by SpA FLSs. We used a membrane-based antibody array for the determination of a large panel of cytokines and chemokines secreted by SpA FLSs stimulated with both TGFβ, TNFα, and IFNγ with or without PFD. A total of 12 cytokines or chemokines had values above the detection limit measured as staining on the membrane array relative to the reference spots. PFD decreased the secretion of insulin-like growth factor-binding protein 3 (IGFBP-3), monocyte-specific chemokine 3 (MCP-3, also called chemokine (C-C motif) ligand 7 (CCL7)), and YKL-40 (also called chitinase-3-like protein 1 (CHI3L1)) more than 2-fold (Fig. 3a and b and Table 1). The changes in secretion of MCP-1 and YKL-40 were validated with ELISA showing similar results. Thus, PFD resulted in no or a modest decrease in MCP-1 secretion and a significant decrease in YKL-40 secretion in SpA FLSs stimulated with both TGFβ, TNFα, and IFNγ (Fig. 3c and d). However, PFD decreased both MCP-1 and YKL-40 in SpA FLSs cultured without stimulation or stimulated with TNFα or IFNγ alone (Fig. 3c and d).

**Table 1:** Effect of PFD on the secretion of cytokines and chemokines from SpA FLSs

Cytokine/chemokine	Fold decrease
**IGFBP-3**	**3.64**
**MCP-3**	**2.88**
***YKL-40***	***2.32***
IL-8	1.30
MIG	1.27
*MCP-1*	*1.23*
Serpin E1	1.21
IL-6	1.13
IP-10	1.08
RANTES	1.04
Trombospondin-1	1.02
ICAM-1	1.02

Using Image StudioTM 4.0 (LI-COR Biosciences UK Ltd) the average pixel density of the duplicate spots was determined along with the three pairs of reference spots on each array. The fold decrease is the ratio of the value of untreated cells divided by the value of pirfenidone treated cells (*n* = 1). IGFBP-3; Insulin-like growth factor-binding protein 3. MCP-3; monocyte-specific chemokine 3 (also called chemokine (C-C motif) ligand 7 (CCL7)). YKL-40 (also called chitinase-3-like protein 1 (CHI3L1)). MIG; monokine induced by gamma interferon (also called chemokine (C-X-C motif) ligand 9 (CXCL9)). Serpin E1 (also called plasminogen activator inhibitor-1 (PAI-1)). IP-10; interferon gamma-induced protein 10 (also called C-X-C motif chemokine 10 (CXCL10)). RANTES; regulated on activation, normal T cell expressed and secreted (also called chemokine (C-C motif) ligand 5 (CCL5)). ICAM-1; Intercellular Adhesion Molecule 1. Bold text and numbers indicate fold change > 2. Italic text and numbers indicate validation with ELISA

### Secretion of bone homeostasis cytokines from SpA FLSs and osteoblast mineralization

**Fig. 4:**
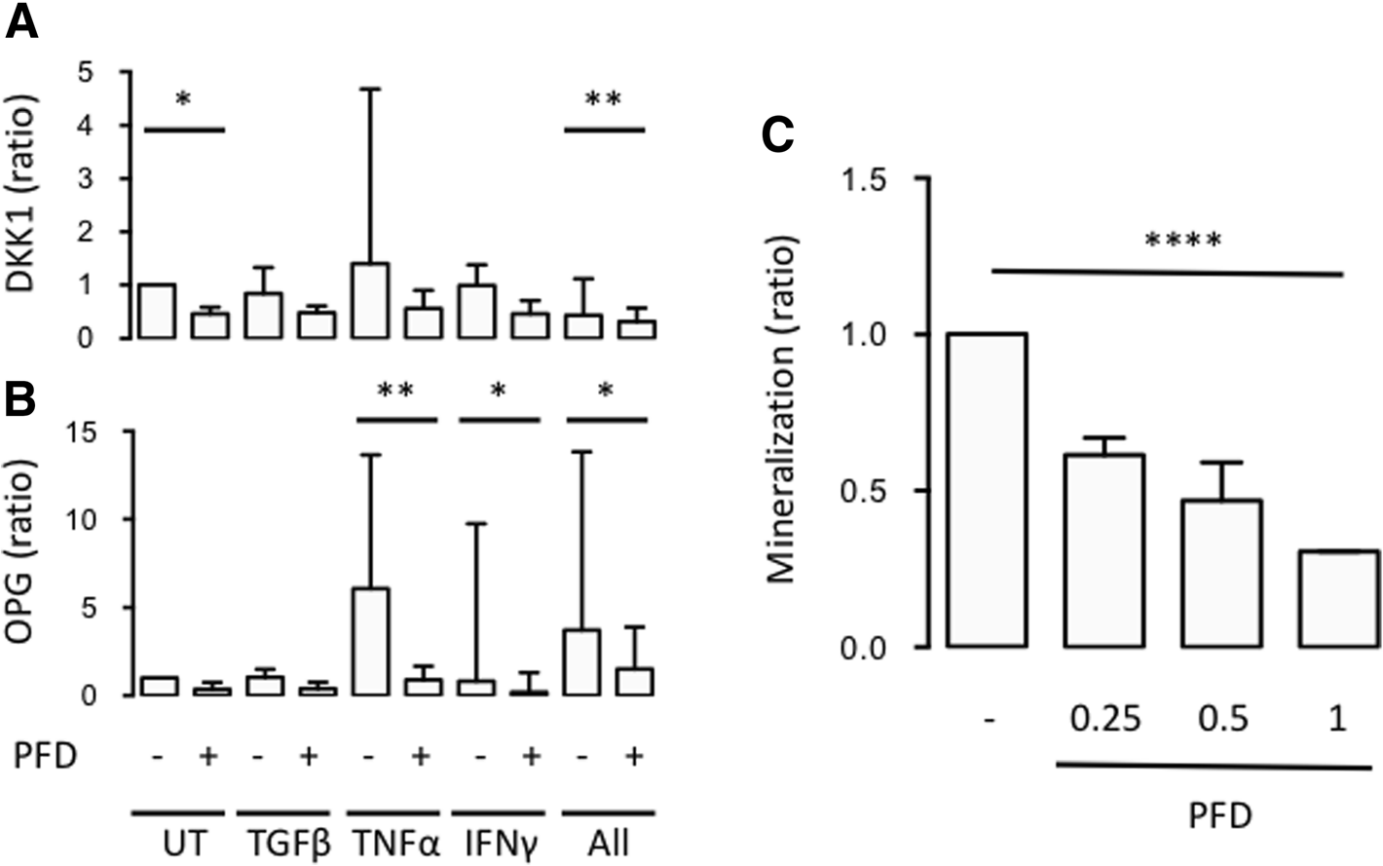
Effects of PFD on secretion of regulators of bone metabolism by SpA FLSs and osteoblast mineralization. **a**-**b** Column bar graph of DKK-1 and OPG secretion by SpA FLSs untreated (UT) and stimulated with TGFβ, TNFα, IFNγ or all three cytokines (All) with or without pirfenidone (PFD) (*n* = 4). Data were normalized to untreated cultures without PFD (ratio), log-transformed and analyzed with the paired t-test. **c** Column bar graph of hydroxyapatite deposition by Saos-2 cells incubated with PFD (triplicates). Saos-2 cells were incubated with increasing concentrations of PFD for 14 days. Data were normalized to untreated cultures without PFD (ratio), log-transformed and comparisons were made with the paired t-test or the repeated measures one-way ANOVA depending on the number of groups. Boxes and bars indicate median and IQR. * *p* < 0.05. ** *p* < 0.01. *** *p* < 0.005

We finally tested whether PFD could interfere with secretion of regulators of bone metabolism by SpA FLSs and osteoblast mineralization. Secretion of the osteoclast activator RANKL, the osteoclast inhibitor OPG, and the osteoblast inhibitor DKK-1 by SpA FLSs was analyzed. PFD decreased the secretion of both DKK1 (*p* = 0.006) and OPG (*p* = 0.02) by SpA FLSs stimulated with a combination of TGFβ, TNFα, and IFNγ (Fig. 4a and b). The concentration of RANKL was below the detection limit of the ELISA assay in all cultures. The mineralization assay was done with Saos-2 cells incubated with increasing concentrations of PFD for 14 days. PFD inhibited the deposition of hydroxyapatite by Saos-2 cells in a dose-dependent manner (*p* = 0.0001) (Fig. 4c).

## Discussion

The pathogenesis of SpA involves both inflammation and new bone formation in the spine. The treatment with non-steroidal anti-inflammatory drugs and inhibitors of TNFα, IL-17 and IL-23 dampens inflammation while new bone formation can progress. Therefore, there is an unmet therapeutic need for the treatment of new bone formation in SpA. Here, we found that PFD inhibits SpA fibroblasts proliferation and cytokine secretion and osteoblast mineralization. Therefore, PFD could be a novel inhibitor of new bone formation in SpA.

In SpA, the purpose of the treatment is to induce clinical remission and prevent radiographic progression. Now, all treatment strategies for SpA are based on suppressing or modulating the immune system, and the agents currently used may induce clinical remission but do not satisfyingly prevent radiographic progression. The radiographic progression is caused by fibrosis and new bone formation. The pathogenesis of fibrosis is poorly understood. TGFβ is the prototypical profibrotic cytokine involved in fibrosis in many organ systems. TGFβ stimulated cells undergo a phenotypical transformation becoming activated myofibroblasts that express contractile proteins such as αSMA. Therefore, the marked upregulation of myofibroblasts in SpA and link to new bone formation is interesting [4, 14, 30, 31]. New bone formation is the result of increased osteoblast mineralization and decreased osteoclast resorption.

PFD exhibits well-documented antifibrotic, anti-inflammatory, and antioxidant activities in a variety of animal and cell-based models, although its molecular target has not been elucidated. In vitro, PFD inhibits the proliferation and activation of a broad variety of cells including fibroblasts, leiomyoma cells, and T cells [15, 16]. In animal models PFD reduces fibrosis in the lung, liver, heart, and kidney [15, 18]. In 2011, PFD was approved for the treatment of idiopathic pulmonary fibrosis [17]. The drug has also been tested in studies with patients with rheumatoid arthritis without serious side effects [19].

In this study, PFD showed both anti-inflammatory and possible anti-fibrotic effects on SpA FLSs. First, the effect of PFD on FLS proliferation and expression of the myofibroblast marker αSMA was studied. PFD inhibited the proliferation of SpA FLSs. Our finding of decreased proliferation in SpA FLSs is in line with previous studies showing inhibition of proliferation of fibroblasts from lung, striated muscle, heart and eye [22, 23, 32, 33]. The effect was seen in both unstimulated cells and in cells under the influence of TGFβ, TNFα, and IFNγ simulating an inflammatory environment. The antiproliferative effect of PFD is not completely understood. PFD has been shown to induce apoptosis in hepatocellular carcinoma cells [34]. In vivo, PFD was found to ameliorate ciclosporine nephrotoxicity by decreasing pro-apoptotic genes and to prevent TNFα induced liver injury [35, 36]. Any effect of PFD on apoptosis of SpA FLS cannot be evaluated from this study. In this study there was no statistically significant suppression of αSMA. However, PFD seemed to have a mild effect to suppress αSMA. This could be interesting because myofibroblast differentiation and formation of extracellular matrix at the entheses seems to be important in SpA [3]. PFD has previously been shown to decrease myofibroblast differentiation and extracellular matrix deposition in fibroblasts from the eye and lung by interfering with the TGFβ signaling pathway [37, 38]. Whether PFD alters myofibroblast formation or extracellular matrix secretion cannot be concluded by this study.

Second, the effect of PFD on SpA FLS expression of membrane molecules and secretion of cytokines and chemokines was studied. Previously, PFD has been shown to inhibit MHC-II molecules in animal models of transplantation [39]. In this study, there was no statistically significant suppression of HLA-DR. However, PFD seemed to have a mild effect to suppress IFNγ induced expression of HLA-DR on SpA FLSs. Therefore, no final conclusions on the effect of PFD on MHC-II expression by SpA FLSs can be made. PFD has also previously been shown to inhibit IL-1β induced expression of ICAM-1 [25]. However, in this study TNFα induced expression of ICAM-1 was not changed in any way by PFD. The discrepancy could be the lower concentrations of PFD used in this study. PFD has also previously been found to decrease the secretion of several cytokines and chemokines such as IL-6, IL-1β, and MCP-1 in animal models of fibrotic disease [18]. This study confirms some of these findings and adds novel targets to the list of inflammatory mediators decreased by PFD. Especially the finding of PFD decreased YKL-40 is interesting because this mediator has been associated with both fibrotic diseases such as pulmonary fibrosis and liver fibrosis and spondyloarthritis [11, 40]. There were large inter-donor variations. Therefore, it is not possible to conclude whether PFD is more effective in preventing MCP-1 or YKL-40 secretion under influence of TGFβ, TNFα, IFNγ or a combination of all three cytokines.

Third, the effect of PFD on osteoblast mineralization and FLS secretion of bone homeostasis proteins was studied. PFD reduced mineralization by osteoblasts. This study is to our knowledge the first to associate PFD with decreased osteoblast activity. In contrast, PFD also tended to decrease DKK-1, which might result in increased osteoblastogenesis. The surmised effect of PFD on osteoblasts in vivo is therefore to be clarified. FLS secretion of OPG, an inhibitor of osteoclastogenesis, was also reduced by PFD. PFD could therefore increase the number of osteoclasts and thus bone resorption. Further studies are needed to validate the effect of PFD on osteoblasts and osteoclasts.

## Conclusion

PFD inhibited SpA FLS proliferation and cytokine production and osteoblast mineralization in vitro. This study encourages studies of the in vivo effect of PFD in SpA.

## Abbreviations

CCL2: Chemokine (C-C motif) ligand 2
CHI3L1: Chitinase-3-like protein 1
DKK1: Dickkopf-related protein 1
FBS: Fetal bovine serum
FLS: Fibroblast-like synovial cell
HC: Healthy control
HLA: Human leukocyte antigen
ICAM: Intercellular adhesion molecule
IFN: Interferon
IL: Interleukin
IQR: Interquartile range
LPS: Lipopolysaccharide
MCP-1: Monocyte chemoattractant protein 1
OPG: Osteoprotegerin
PBS: Phosphate buffered saline
PFD: Pirfenidone
RANK: Receptor activator of nuclear factor-κB
SFMC: Synovial fluid mononuclear cell
SMA: Smooth muscle actin
SpA: Spondyloarthrtis
TGF: Transforming growth factor
TNF: Tumour necrosis factor
UT: Untreated
YKL40 (CHI3L1): Chitinase-3-like protein 1
